# *Lycium barbarum* Polysaccharides Improved Glucose Metabolism in Prediabetic Mice by Regulating Duodenal Contraction

**DOI:** 10.3390/nu15204437

**Published:** 2023-10-19

**Authors:** Doudou Li, Xiaoke Zhang, Yanna Fan, Yannan Zhang, Xiujuan Tao, Jianjun Yang

**Affiliations:** 1 School of Public Health, Ningxia Medical University, Yinchuan 750004, China; m15202684476@163.com (D.L.); m18892028816@163.com (X.Z.); nananeimeng@163.com (Y.F.); yannan_jn@163.com (Y.Z.); taotaonxykd@163.com (X.T.); 2 Ningxia Key Laboratory of Environmental Factors and Chronic Disease Control, Yinchuan 750004, China

**Keywords:** prediabetes, *Lycium barbarum* polysaccharides, gut microbiota, short-chain fatty acid, blood glucose

## Abstract

*Lycium barbarum* polysaccharides (LBPs) have been shown to exert an antiglycemic effect. Emerging evidence suggests that patients with hyperglycemia have a hypercontractility of duodenum, and targeting duodenal contraction of duodenum can be beneficial to glucose metabolism. However, it is unknown whether LBPs can improve glucose metabolism by regulating the hypercontractility of the duodenum. Our aim was to explore the effect of LBPs on duodenal contraction in prediabetic mice and also preliminarily investigate the mechanism. The results showed that LBPs improved glucose homeostasis by decreasing the duodenal amplitude of contraction rather than frequency. Moreover, LBPs ameliorated the gut microbiota composition and the levels of short-chain fatty acids, especially acetic acid, which might bind to the receptor on neurons to regulate the contraction of the duodenum. Acetic acid was hypothesized to play a key role in the above process. Then, acetic acid was determined to exert an antiglycemic effect as expected. In conclusion, LBPs may rely on acetic acid to regulate duodenal contraction to ameliorate glucose metabolism in prediabetic mice, which provides a new therapeutic strategy to treat dysglycemia.

## 1. Introduction

Prediabetes is the earliest stage in the natural history of diabetes and includes impaired fasting glucose (IFG) and impaired glucose tolerance (IGT). It is estimated that 11.4% of adults around the world will have IGT, and 6.9% will have IFG by 2045 [1]. Surprisingly, a cross-sectional study in mainland China showed that the prevalence of prediabetes was 35.7% in 2013 and 38.1% in 2018 in estimation [2]. Individuals with prediabetes are more likely to develop diabetes and cardiovascular diseases. Studies have demonstrated that prediabetes is actually a high-risk state for diabetes without intervention [3]. In a nested case-control study in Japan, people with prediabetes were reported to be at risk of cardiovascular diseases [4]. The concept of prediabetes is to identify individuals with abnormal blood glucose levels and support them to take action to maintain health.

The gut–brain axis refers to the connection between the gastrointestinal system and the brain [5]. It has been reported that the gut–brain axis is involved in the regulation of glucose homeostasis [6]. The gut can sense glucose via different receptors, which are mainly expressed in enterocytes, brush cells, enteroendocrine cells and enteric neurons in the small intestine. Neural and endocrine signals from the gut are sent to the hypothalamus for the detection of glucose fluctuations. After receiving this information, the hypothalamus sends signals to peripheral tissues via the autonomous nervous system to maintain glucose homeostasis [7]. However, individuals with abnormal glucose levels have been reported to have intestinal hypercontractility [8], breaking the signal between the gut and the brain. Recently, studies have demonstrated that decreasing duodenal contraction is a novel therapeutic strategy for dysglycemia [9,10]. The movement of the small intestine is mainly regulated by the enteric nervous system (ENS) [11], which reduces intestinal contraction by releasing inhibitory neurotransmitters, such as nitric oxide (NO), and stimulates intestinal contraction by excitatory neurotransmitters, such as acetylcholine [12].

As one of the well-known theories of traditional Chinese medicine, the theory of medicine and food homology, according to which many foods can also be used as medicines themselves, has gradually become prominent. Accumulating studies have revealed the benefits of homologous medicinal and edible foods on glucose metabolism, including *Lycium barbarum* [13,14]. Among bioactive components of *Lycium barbarum*, polysaccharides are thought to be effective in lowering blood glucose [15]. LBPs are heteropolysaccharides that comprise seven monosaccharides [16]. A four-week LBP treatment ameliorated glucose metabolism and insulin resistance in NASH rat models [17]. Li found that LBPs decreased the levels of antioxidant enzymes, thereby protecting the liver and kidneys of diabetic rats [18]. However, whether LBPs can improve blood glucose levels by regulating duodenal hypercontractility remains unclear.

There is considerable evidence that the gut microbiota and metabolites are closely implicated in the development of diseases, such as type 2 diabetes mellitus (T2DM), obesity, nonalcoholic fatty liver disease and cancer [19]. Consequently, treatments targeting the gut microbiota and related pathways have been widely discussed [20]. In previous work, we found that LBPs affected microbiota composition and short-chain fatty acids (SCFAs) concentration in Sprague-Dawley rats [21]. Free fatty acid receptor (FFA) 3, a SCFA receptor, is expressed in enteric neurons, releasing acetylcholine and activation of FFA3 can inhibit acetylcholine secretion [22], but whether the gut microbiota and its metabolites are involved in the regulation of duodenal hypercontractility by LBPs warrants further investigation.

In order to investigate whether LBPs are dependent on duodenal contraction to ameliorate glucose metabolism, we evaluated the efficacy as well as duodenal amplitude and frequency of contraction after LBP treatment in comparison with prediabetic mice. The mechanism of LBPs in the improvement of duodenal contraction by modulating gut microbiota and metabolites deserves further exploration. Our study would provide novel perspectives on the treatment of dysglycemia.

## 2. Materials and Methods

### 2.1. Materials and Reagents

LBPs (B20460) were purchased from Shanghai Yuanye Biological Technology Co., Ltd. (Shanghai, China). The characterization of LBPs was analyzed as previously described [21]. Sodium acetate was purchased from Yantai Shuangshuang Chemical Co., Ltd. (Yantai, China). A normal fat diet (NFD) was provided by Beijing Keao Xieli Feed Co., Ltd. (Beijing, China) and a high-fat diet (HFD) containing 45% fat was provided by Research Diets, Inc. (New Brunswick, NJ, USA). Triglycerides (TG), total cholesterol (TC), low-density lipoprotein cholesterol (LDL-C), high-density lipoprotein cholesterol (HDL-C) and insulin assay kits were supplied by Nanjing Jiancheng Bioengineering Institute (Nanjing, China). The glucometer was obtained from ACON Biotech (Hangzhou) Co., Ltd. (Hangzhou, China). Ampicillin, neomycin sulfate and metronidazole were obtained from Solarbio Science and Technology (Beijing) Co., Ltd. (Beijing, China), and vancomycin was obtained from Shanghai Macklin Biochemical Technology Co., Ltd. (Shanghai, China).

### 2.2. Experimental Protocol

C57BL/6J mice were purchased from the Experimental Animal Center of Ningxia Medical University. All mice were housed under specific pathogen-free conditions and had free access to water and food. They were maintained on a 12 h light/12 h dark cycle.

Experiment 1: Nine-week-old male mice were randomly divided into the following five groups (*n* = 7 mice per group): (1) NFD, (2) HFD, (3) HFD + LBPs-L (high-fat diet with 50 mg/kg LBPs), (4) HFD + LBPs-M (high-fat diet with 100 mg/kg LBPs) and (5) HFD + LBPs-H (high-fat diet with 150 mg/kg LBPs). The mice were fed their respective diets for 12 weeks. NFD-treated mice were orally administered drinking water, and 0, 50, 100 and 150 mg/kg LBPs were administered to HFD-treated mice for 12 weeks.

Experiment 2: Five-week-old male mice were treated with an antibiotic cocktail (ABx; ampicillin 1 g/L, neomycin sulfate 1 g/L, metronidazole 1 g/L and vancomycin 0.5 g/L) in drinking water for 4 weeks to deplete the gut microbiota [23]. Mice were randomly divided into the following two groups (*n* = 7 mice per group): (1) ABx and (2) ABx + LBPs-H (antibiotic cocktail with 150 mg/kg LBPs). The ABx group was orally administered drinking water, and 150 mg/kg LBPs were orally administered to ABx + LBPs-H mice for 12 weeks. Mice were fed a HFD and treated with drinking water containing ABx for 16 weeks.

Experiment 3: Nine-week-old male mice were randomly divided into the following two groups (*n* = 7 mice per group): (1) HFD and (2) HFD + acetic acid (280 mg/kg). Mice were fed a HFD for 12 weeks. Mice in the HFD group were orally administered drinking water, and mice in the HFD + acetic acid group were administered 280 mg/kg sodium acetate for 12 weeks.

### 2.3. Oral Glucose Tolerance Test (OGTT) and Fasting Blood Glucose (FBG)

Mice had fasted overnight and then were given 3 g/kg body weight of glucose. Blood was collected at 0, 15, 30, 60, 90 and 120 min from the tail vein. The area under the curve (AUC) was calculated to evaluate the results of OGTT. The value of blood glucose at 0 min was FBG.

### 2.4. Biochemical Assays and Enzyme-Linked Immunosorbent Assay (ELISA)

Orbital blood was collected into a 1.5 mL centrifuge tube and centrifuged (3000 rpm at 4 °C) later. After 15 min, the serum at the top was collected and stored in the refrigerator at −80 °C. The levels of TG, TC, LDL-C and HDL-C were determined following instructions. Fasting serum insulin (FINS) levels were measured with an insulin assay kit.

### 2.5. Measurement of Duodenal Contraction

The duodenum was removed immediately after the mouse was euthanized. Then, it was incubated with an oxygenated Tyrode’s Solution (NaCl 8 g/L, KCl 0.2 g/L, CaCl_2_ 0.2 g/L, MgCl_2_ 0.2 g/L, NaHCO_3_ 1 g/L, KH_2_PO_4_ 0.05 g/L and glucose 1 g/L), attached to the isotonic transducer (BL-420N biological signal acquisition and analysis system, Chengdu Techman Software Co., Ltd., Chengdu, China). Isotonic contractions were recorded following transducer displacement for 10 min.

### 2.6. Analysis of Cecal Microbiota Composition

The cecal contents of mice in the NFD, HFD and HFD + LBPs-H groups were collected and stored at −80 °C. The total DNA was extracted by a QIAamp DNA Stool Mini Kit (Qiagen, Valencia, CA, USA). The V3-V4 region of bacterial 16S rDNA was amplified. The DNA was sequenced by the Illumina MiSeq platform, and microbiota composition was analyzed by QIIME 2.0. Sequences with ≥97% similarity were grouped into identical OTUs. The unweighted UniFracBeta distance was applied to a principal coordinate analysis (PCoA), and the unweighted pair group method with arithmetic mean (UPGMA) clustering, complex and multidimensional data was obtained and visualized in master coordinates by the PCoA. A variation ranging from the distance matrix to a new set of orthogonal axes is required for this type of analysis. The first principal coordinate was used to denote the maximum variation factor, and the second principal coordinate was used to denote the second maximum variation factor, and so on.

### 2.7. SCFAs Quantification Analysis

An appropriate amount of samples was added to 500 μL diluted water and homogenized for 1 min. The supernatant (200 μL) was collected into a tube after the sample was centrifuged at 12,000× *g* rpm for 10 min at 4 °C. Next, the supernatant was mixed with 100 μL of 15% phosphoric acid, 20 μL of internal standard isocaproic acid and 280 μL of diethyl ether. The mixture was homogenized for 1 min and centrifuged at 12,000× *g* rpm for 10 min at 4 °C. At last, the supernatant was collected for further analysis. The SCFA concentration was determined by Thermo Trace 1310 gas chromatography and Thermo ISQ LT mass spectrometry.

### 2.8. Statistical Analysis

The data were analyzed using a one-way analysis of variance (ANOVA) among groups and an unpaired two-tailed Student’s *t*-test between two groups using GraphPad Prism 9 (GraphPad Software, San Diego, CA, USA). Results were considered significantly different at *p* < 0.05. Data are presented as mean ± standard deviation (SD).

## 3. Results

### 3.1. LBPs Improved Glucose Homeostasis in Prediabetic Mice

**Figure 1:**
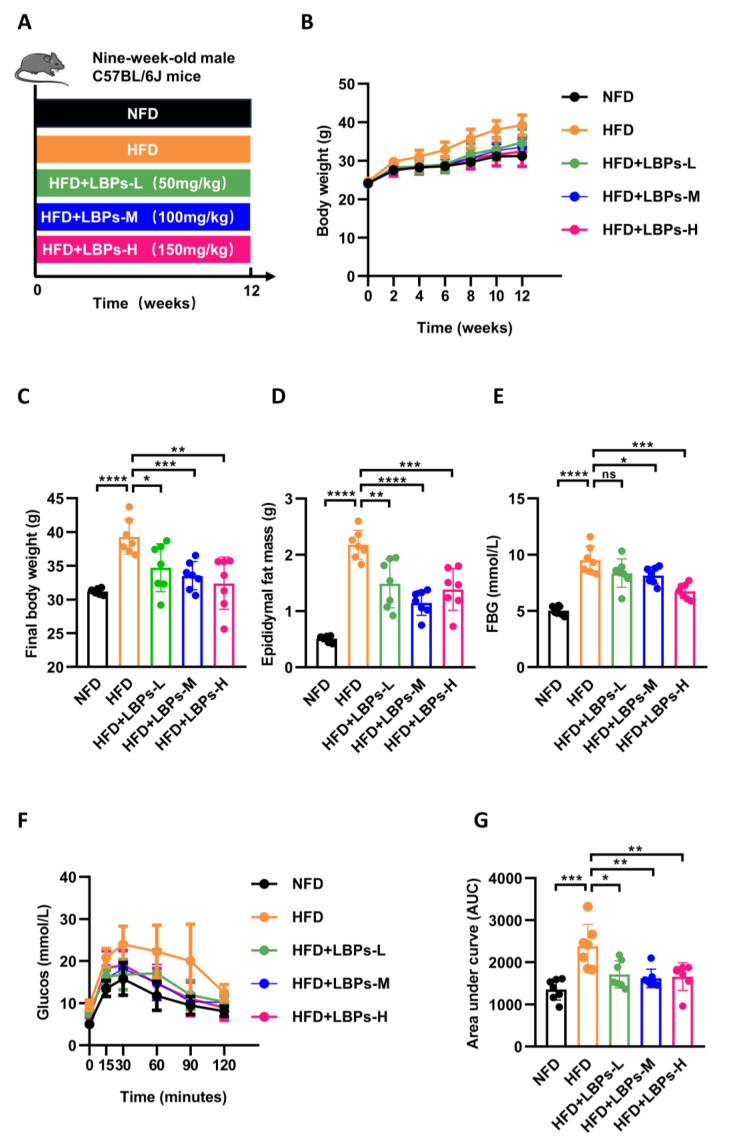

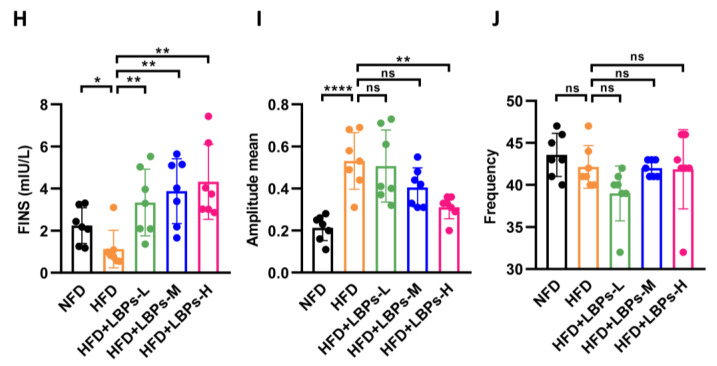
Effects of LBPs on glucose homeostasis and duodenal contraction in prediabetic mice. (**A**) Design of experiment 1. (**B**) Body weight during 12 weeks. (**C**) Final body weight. (**D**) Epididymal fat mass. (**E**) FBG. (**F**) The result of OGTT. (**G**) The AUC of OGTT. (**H**) FINS. (**I**) The duodenal amplitude of contraction. (**J**) The duodenal frequency of contraction. * *p* < 0.05; ** *p* < 0.01; *** *p* < 0.001; **** *p* < 0.0001; ns, not statistically significant.

Body weight gain was greater in HFD-fed mice than in NFD mice. LBP treatment decreased the HFD-induced increase in body weight, particularly with high-dose LBPs (Figure 1B). The final body weight of the HFD group was 25.87% higher than that of the NFD group, and medium and high doses of LBPs reduced the body weight of HFD-treated mice by 14.62% and 17.49%, respectively (Figure 1C). HFD-fed mice showed an increase in epididymal fat mass compared with NFD. Different doses of LBPs reduce epididymal fat mass, with the medium dose having the most significant effect (Figure 1D).

To determine the effects of LBPs on glucose homeostasis, we tested FBG and FINS levels (Figure 1E,H). The FBG levels of mice in the HFD group were higher than those in the NFD group, and the FINS levels in the HFD group were lower than those in the NFD group. Administration of medium and high doses of LBPs significantly reduced FBG and increased FINS levels compared with the HFD. However, mice in the LBPs-L group show only a statistical increase in FINS in comparison with mice in the HFD group. An OGTT was also performed, and all doses of LBPs improved glucose tolerance in HFD mice (Figure 1F,G).

In terms of lipid metabolism, the levels of TC, LDL-C and LDL-C/HDL-C were high in HFD mice after 12 weeksbut were obviously improved by LBP administration (Table 1). Additionally, the HDL-C levels were significantly higher in the LBPs-L and LBPs-M groups than in the HFD group. 

As shown in Figure 1G,H, 12-week HFD led to a remarked increase in the duodenal amplitude of contraction compared with the 12-week NFD but no increase in frequency. The duodenal amplitude of contraction in the LBPs-H group was decreased than that in the HFD group, which demonstrates that high-dose LBPs can decrease hypercontractility of duodenum. In contrast, low and medium doses of LBPs failed to exert influence on the duodenal amplitude of contraction. These results illustrated that LBPs produced an antiglycemic effect and modulated the duodenal amplitude of contraction of prediabetic mice.

### 3.2. LBPs Regulated Duodenal Contraction via Gut Microbiota to Improve Glucose Homeostasis in Prediabetic Mice

**Figure 2:**
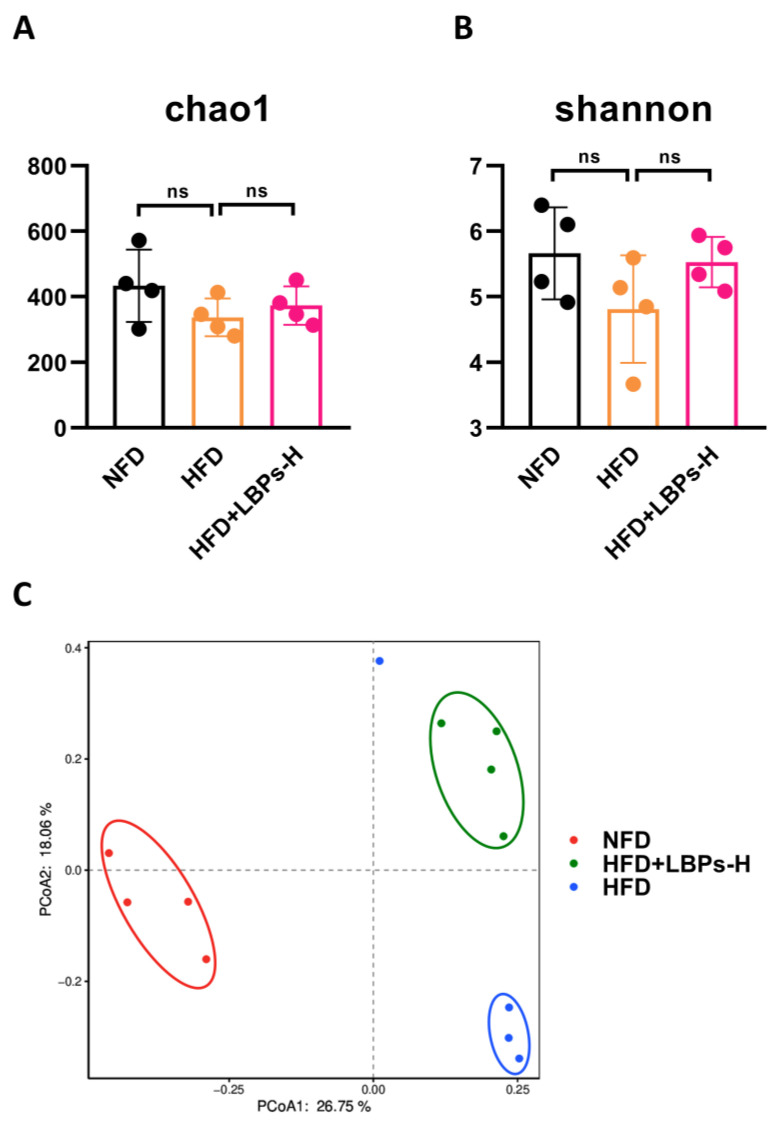

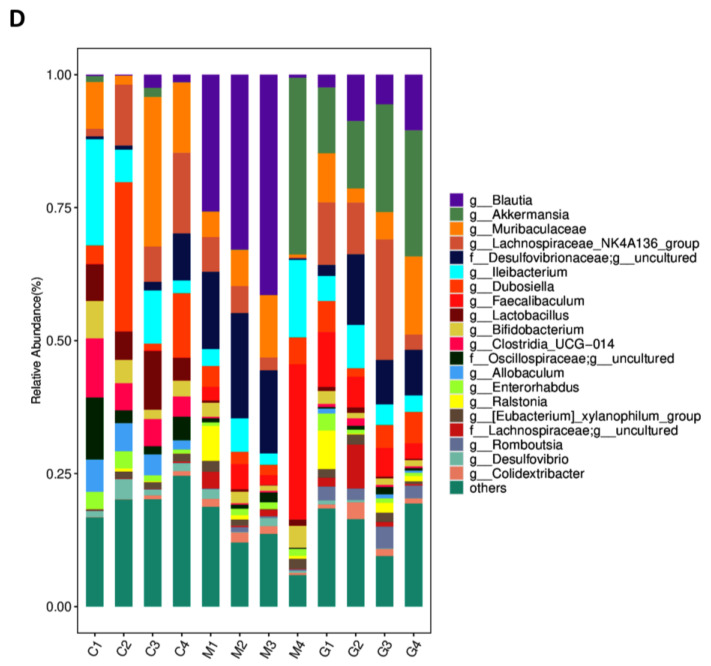
Effects of LBPs on gut microbiota composition in prediabetic mice. (**A**) chao1 indices. (**B**) Shannon indices. (**C**) The result of PCoA. (**D**) The composition of gut microbiota on genus level. ns, not statistically significant.

The alpha diversity of the gut microbiota can be expressed by the chao1 index and Shannon index, which indicate species richness. There was no significant difference between the HFD group and LBPs-H groups; however, there was an increasing tendency in the LBPs-H group (Figure 2A,B). There were obvious differences among the three groups, and the LBPs-H group was separated from the HFD group, indicating that the microbial community structure of HFD-mice has changed after high-dose LBP treatment (Figure 2C). The gut bacterial profiles of the three groups were analyzed at the genus level. The relative abundances of *Akkermansia* and *Lachnospiraceae_NK4A136_group* were higher in the LBPs-H group compared with the HFD group (Figure 2D).

**Figure 3:**
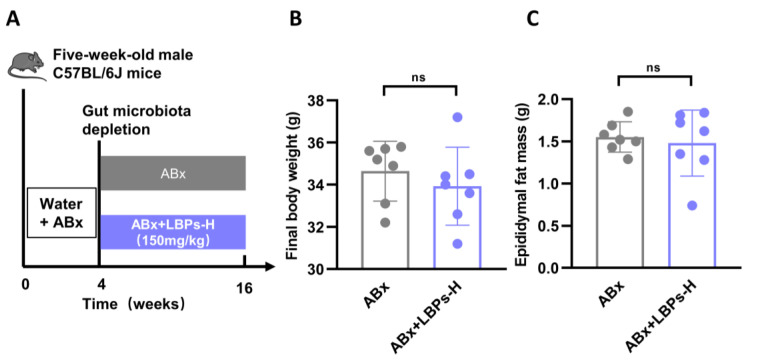

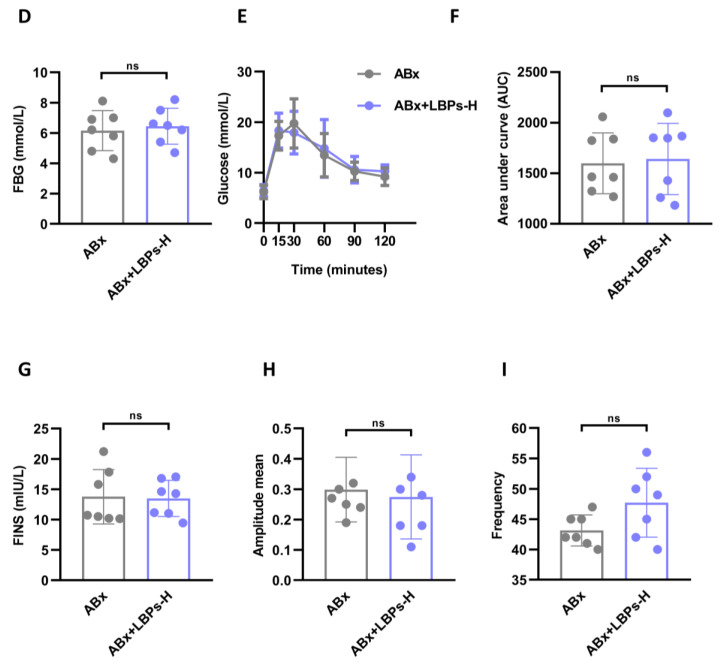
Effects of high dose of LBPs on glucose metabolism in ABx-treated mice. (**A**) Design of experiment 2. (**B**) Final body weight. (**C**) Epididymal fat mass. (**D**) FBG. (**E**) The result of OGTT. (**F**) The AUC of OGTT. (**G**) FINS. (**H**) The duodenal amplitude of contraction. (**I**) The duodenal frequency of contraction. ns, not statistically significant.

To determine whether the gut microbiota is involved in the regulation of duodenal hypercontractility by LBPs, we treated HFD mice with ABx supplemented with or without high-dose LBPs. Figure 3 and Table 2 showed that there were no statistical differences between the two groups, indicating that the LBP treatment abolished beneficial effects on body weight, epididymal fat mass, FBG, INS, glucose tolerance, lipid metabolic indices and duodenal amplitude of contraction after the gut microbiota had been depleted. These results indicate that the gut microbiota may be required for the antiglycemic effects of LBPs by modulating duodenal contraction.

### 3.3. LBPs Regulated Duodenal Contraction via Acetic Acid to Improve Glucose Homeostasis in Prediabetic Mice

**Figure 4:**
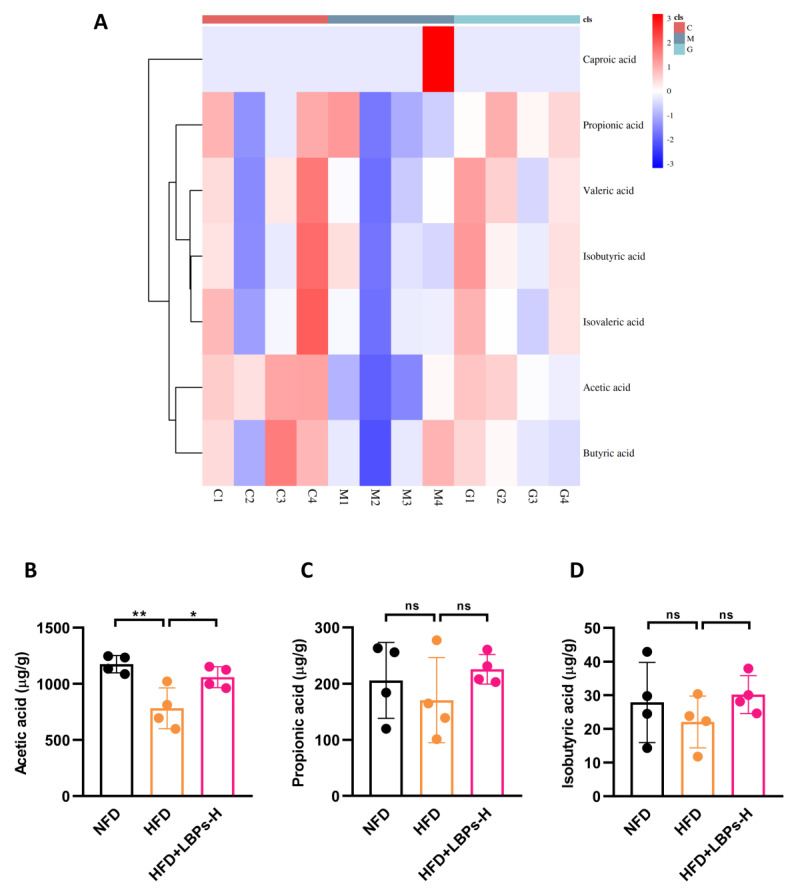

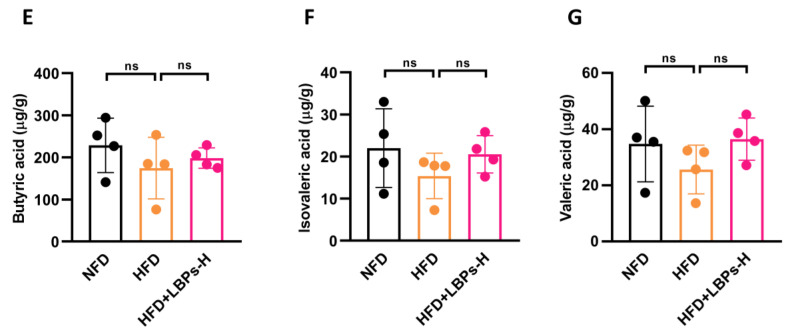
Effects of high dose of LBPs on different kinds of SCFAs in prediabetic mice. (**A**) The heatmap of SCFAs. (**B**) Acetic acid. (**C**) Propionic acid. (**D**) Isobutyric acid. (**E**) Butyric acid. (**F**) Isovaleric acid. (**G**) Valeric acid. * *p* < 0.05; ** *p* < 0.01; ns, not statistically significant.

SCFAs have been reported to play vital roles in glucose metabolism [24]; therefore, we also performed SCFAs quantification analysis (Figure 4). Compared with the NFD group, the acetic acid levels in the HFD group were greatly decreased. However, the acetic acid levels were elevated after 12-week supplementation with LBPs (Figure 4B). There were no significant differences in other types of SCFAs among the three groups (Figure 4C–G). These results confirmed that acetic acid was involved in the regulation of duodenal hypercontractility by LBPs.

**Figure 5:**
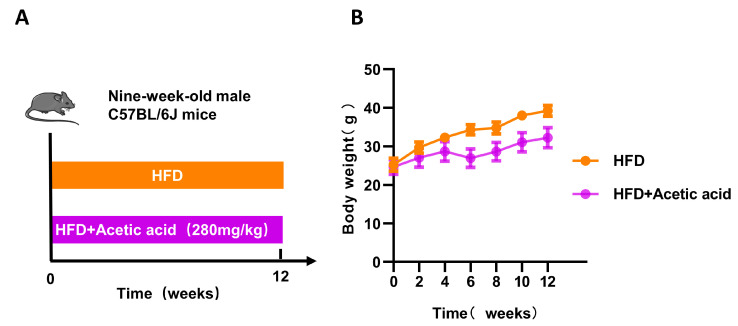

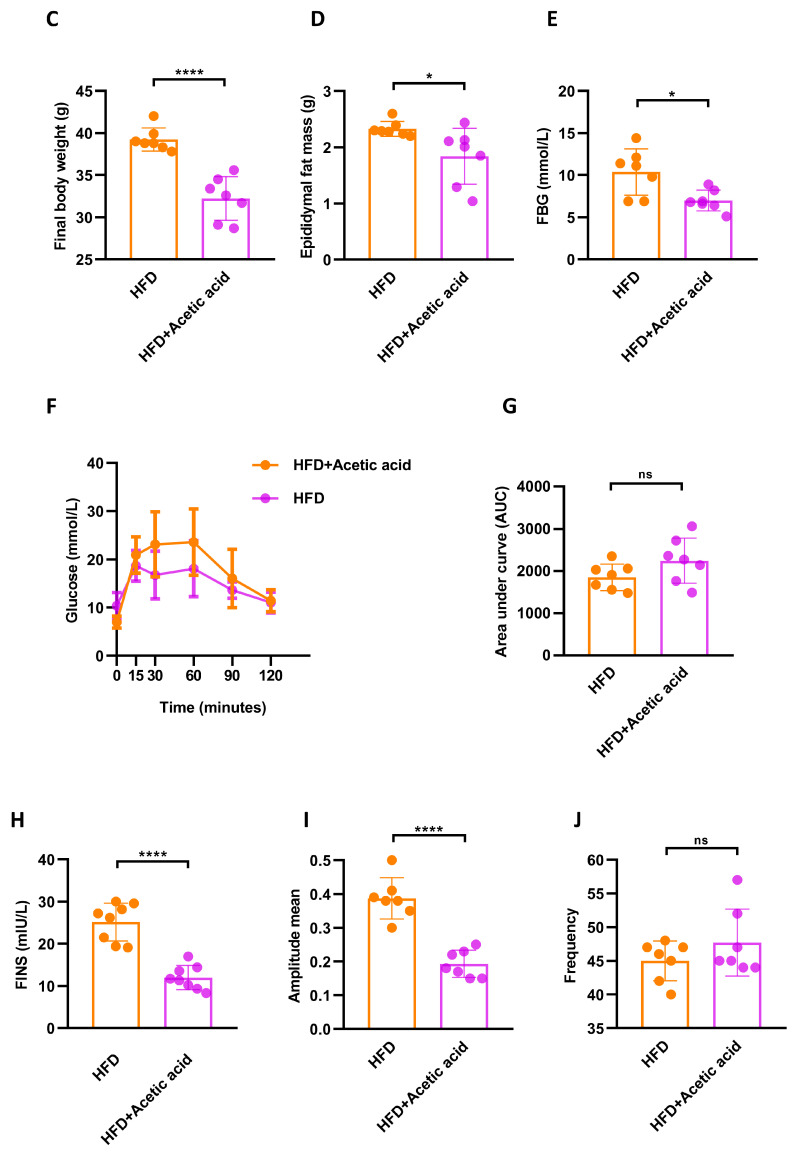
Effects of acetic acid on glucose homeostasis in prediabetic mice. (**A**) Design of experiment 3. (**B**) Body weight during 12 weeks. (**C**) Final body weight. (**D**) Epididymal fat mass. (**E**) FBG. (**F**) The result of OGTT. (**G**) The AUC of OGTT. (**H**) FINS. (**I**) The duodenal amplitude of contraction. (**J**) The duodenal frequency of contraction. * *p* < 0.05; **** *p* < 0.0001; ns, not statistically significant.

Then, 9-week-old HFD mice were treated with or without oral gavage of acetic acid for 12 weeks. HFD led to a greater increase in body weight compared with that in NFD in mice, and LBP treatment reduced body weight (Figure 5B). In addition, the mice fed a HFD supplemented with acetic acid produced a statistical decrease in final body weight, epididymal fat mass, FBG and INS and an increase of HDL-C in comparison with mice fed an HFD (Figure 5C–E,H and Table 3). However, acetic acid did not show significant improvement in glucose tolerance (Figure 5F,G). In terms of duodenal contraction, supplementation with acetic acid obviously decreased the amplitude of contraction but had no effect on the frequency of contraction relative to the HFD group (Figure 5I,J). These results suggested that acetic acid might be a key factor in the antiglycemic effect of LBPs by regulating duodenal contraction.

## 4. Discussion

In our present work, we preliminarily explored the mechanism by which LBPs improved glucose metabolism in prediabetic mice. LBPs decreased the hypercontractility of the duodenum in prediabetic mice to improve glucose metabolism by modulating the gut microbiota and their metabolite acetic acid. Acetic acid might bind to the SCFAs receptors on enteric neurons, releasing acetylcholine, thereby decreasing the hypercontractility of the duodenum and restoring the connection between the gut and brain.

Due to its substantial prevalence, prediabetes is considered to be a major challenge for diabetes across the world [1]. Lifestyle modifications (diet and exercise) and drug therapies have been suggested for the treatment of prediabetes [25]. According to the Chinese Da Qing study in which people with impaired glucose tolerance were followed up for 30 years, subjects assigned to the combined intervention group (diet and exercise) showed delayed onset of T2DM by 3.96 years, decreased the incidence of complications and prolonged life expectancy in comparison with subjects in the control group [26]. Among several drugs, metformin has been used for the management of prediabetes because of its hypoglycemic action, low cost and efficacy in clinical trials [27]. However, Davidson did not agree with this opinion for several reasons [28]. As it is difficult for individuals to maintain lifestyle modifications and there is no consensus on drug therapies, new treatments to improve prediabetes are still needed nowadays. Studies have shown that phytogenic substances are beneficial for glucose metabolism [29], and LBPs seem to be quite a good choice [30]. The hypoglycemic effects of LBPs have been demonstrated in animal experiments and clinical trials [17,31]. Consistent with other studies, we also determined the hypoglycemic effect of LBPs in prediabetic mice. In our study, prediabetic mice showed significantly decreased FBG and increased FINS levels, but also improved glucose tolerance after LBPs intervention (*p* < 0.05). In addition, LBPs positively regulated lipid metabolism. The 12-week HFD treatment led to an obvious increase in body weight and epididymal fat mass, which decreased after LBPs supplementation.

Glycometabolic disorders were improved by LBPs in our study, and we explored the underlying mechanism. At present, some mechanisms have been proposed to explain the effects of LBPs. Oxidative stress has been reported to be closely associated with β-cell dysfunctions as well as insulin resistance [32,33]. LBPs were considered to be antioxidant agents, for they can increase levels of superoxide dismutase and glutathione and decrease levels of malondialdehyde [34]. Yang et al. found that LBPs activated nuclear factor-E2-related factor 2 (Nrf2), a key factor in the antioxidant system, by HepG2 cells and HFD-fed mice [35]. In addition, LBPs can improve insulin resistance, which is responsible for the development of dysglycemia. LBPs have been reported to increase the translocation of glucose transporter 4 (GLUT4), the main glucose transporter in skeletal muscle, from the intracellular pool to the cell surface, thereby facilitating glucose uptake [36]. Furthermore, p38 mitogen-activated protein kinase (p38 MAPK) and phosphoinositol 3-kinase (PI3-K) were demonstrated to be key factors for the translocation and activation of GLUT4 [37]. Using STC1 cells and diabetic KKAy mice, glucagon-like peptide 1 (GLP1) was found to be increasingly secreted after administration of LBPs, for calcium ion influx was elevated, and alpha-glucosidase was inhibited in the first phase secretion of GLP1 and Gcg gene was regulated in the second phase [38]. In addition, LBPs improve the quantity and function of β cells, which contribute to the secretion of insulin [39]. Our results showed that LBPs regulated duodenal contraction to improve glucose homeostasis. In recent years, targeting the ENS/contraction of the duodenum has been a novel therapeutic strategy for disorders of glucose metabolism [9,40]. On this basis, our study revealed that the duodenal amplitude of contraction could be obviously decreased in prediabetic mice supplemented with LBPs, restoring the communication between the gut and the brain.

Dysbiosis of the gut microbiome is associated with glucose metabolism [41]. By bidirectional Mendelian randomization (MR) analyses, microbiota profiles have been demonstrated to determine the risk of developing T2DM [42]. Meanwhile, the feature of disrupted gut bacterial rhythmicity can also predict the risk of the onset of T2DM [43]. Previous studies have shown that gut microbiota and metabolites have been successful in modulating host glucose metabolism [20]. Then, we examined the gut microbiota of mice in our study. Because high-dose LBPs significantly decreased hypercontractility of the duodenum, we analyzed gut microbiota in the LBPs-H group rather than the LBPs-L group and LBPs-M group. Results have shown that the administration of LBPs can increase the relative abundance of Akkermansia and Lachnospiraceae_NK4A136_group at the genus level. *Akkermansia muciniphila* (*A. muciniphila*) is one of the species of Akkermansia. Administration of *A. muciniphila* to HFD-mice induced GLP-1 expression [44], and metabolic parameters were improved in overweight/obese insulin-resistant subjects supplemented with pasteurized *A. muciniphila* [45]. Lachnospiraceae plays pivotal roles in human health and can produce abundant SCFA, including butyrate and acetic acid [46]. Lachnospiraceae were increased in inulin-treated diabetic mice [47]. As can be seen, Akkermansia and Lachnospiraceae were beneficial to glucose metabolism, which was already confirmed in our study. Furthermore, the depletion of gut microbiota failed to decrease duodenal hypercontractility and abolished the anti-hyperglycemic effect of LBPs on HFD mice in our study, illustrating that LBPs relied on gut microbiota to regulate duodenal contraction to improve glucose homeostasis.

Among the different SCFAs, only the level of acetic acid was significantly elevated after LBP supplementation in our study. Previous studies have shown that taking 10–30 mL of vinegar daily is beneficial to glucose metabolism [48]. The receptor of SCFA was expressed on the enteric neurons [22]. Therefore, we hypothesized that administration of LBPs to mice would improve intestinal microecology and increase the level of acetic acid. Thus, acetic acid activates the receptors on enteric neurons, which inversely regulates acetylcholine secretion, thereby decreasing duodenal hypercontractility and restoring the connection between the gut and brain. Based on this assumption, we treated HFD mice with acetic acid for 12 weeks. Our results showed that acetic acid effectively reduced body weight, which was consistent with results in human and animal studies [49]. Another study also found that the serum level of acetic acid was negatively correlated with obesity [50]. Epididymal fat mass can be decreased after acetic acid treatment, and other studies have shown that supplementation of acetate helps reduce fat accumulation [51]. In our study, acetic acid obviously lowered FBG levels and decreased the duodenal amplitude of contraction, which confirmed our hypothesis that LBPs regulate duodenal contraction via acetic acid to improve glucose homeostasis. It has been demonstrated that acetic acid can improve glucose tolerance and lipid disorders [52,53]; however, these results were not observed in our study, probably due to the dosage of acetic acid.

## 5. Conclusions

In conclusion, our study illustrated the beneficial effects of LBPs on glucose metabolism in prediabetic mice. LBPs improved the gut microbiota composition and SCFA concentrations, especially that of acetic acid. Acetic acid might bind to the receptors on enteric neurons to modulate the hypercontractility of the duodenum, thereby ameliorating glucose homeostasis.

## Figures and Tables

**Table 1:** Effect of different doses of LBPs treatment on serum lipid levels.

Parameters	NFD	HFD	HFD + LBPs-L	HFD + LBPs-M	HFD + LBPs-H
TG (mmol/L)	1.08 ± 0.12	1.07 ± 0.15	1.28 ± 0.19 *	1.04 ± 0.27	0.99 ± 0.19
TC (mmol/L)	2.83 ± 0.37	6.61 ± 0.93 ^####^	3.92 ± 0.84 ***	5.01 ± 0.35 **	4.74 ± 1.03 **
HDL-C (mmol/L)	6.44 ± 2.63	5.46 ± 0.44	3.36 ± 0.92 ***	3.90 ± 0.58 ****	5.48 ± 0.71
LDL-C (mmol/L)	0.15 ± 0.04	1.23 ± 0.31 ^####^	0.43 ± 0.21 ***	0.34 ± 0.14 ****	0.46 ± 0.21 ***
LDL-C/HDL-C	0.02 ± 0.01	0.23 ± 0.07 ^####^	0.13 ± 0.06 *	0.08 ± 0.03 ***	0.08 ± 0.04 ***

^####^ *p* < 0.0001 compared to the NFD group; * *p* < 0.05, ** *p* < 0.01, *** *p* < 0.001 and **** *p* < 0.0001 compared to the HFD group. Data are expressed as mean ± standard deviation.

**Table 2:** Effect of high dose of LBPs treatment on serum lipid levels.

Parameters	ABx	ABx + LBPs-H
TG (mmol/L)	1.66 ± 0.27	0.75 ± 0.31 ****
TC (mmol/L)	2.86 ± 0.26	2.60 ± 0.43
HDL-C (mmol/L)	1.77 ± 0.38	1.31 ± 0.34 *
LDL-C (mmol/L)	23.99 ± 3.66	20.92 ± 5.41
LDL-C/HDL-C	14.21 ± 4.00	16.65 ± 5.52

* *p* < 0.05 and **** *p* < 0.0001 compared to the ABx group. Data are expressed as mean ± standard deviation.

**Table 3:** Effect of acetic acid treatment on serum lipid levels.

Parameters	HFD	HFD + Acetic Acid
TG (mmol/L)	1.43 ± 0.28	1.17 ± 0.22
TC (mmol/L)	3.84 ± 0.75	3.47 ± 0.67
HDL-C (mmol/L)	1.73 ± 0.36	2.58 ± 0.84 *
LDL-C (mmol/L)	49.65 ± 7.75	38.04 ± 11.87
LDL-C/HDL-C	29.53 ± 7.07	17.44 ± 10.73 *

* *p* < 0.05 compared to the HFD group. Data are expressed as mean ± standard deviation.

## Data Availability

Data are available from the corresponding authors upon reasonable request.
